# Wood Cellular Dendroclimatology: Testing New Proxies in Great Basin Bristlecone Pine

**DOI:** 10.3389/fpls.2016.01602

**Published:** 2016-10-25

**Authors:** Emanuele Ziaco, Franco Biondi, Ingo Heinrich

**Affiliations:** ^1^DendroLab, University of Nevada, RenoNV, USA; ^2^GFZ German Research Centre for GeosciencesPotsdam, Germany; ^3^Institute of Geography, Humboldt-UniversityBerlin, Germany

**Keywords:** wood anatomy, palaeoclimatic reconstruction, multi-proxy, tree-rings, lumen diameter, climatic variability, conifers

## Abstract

Dendroclimatic proxies can be generated from the analysis of wood cellular structures, allowing for a more complete understanding of the physiological mechanisms that control the climatic response of tree species. Century-long (1870–2013) time series of anatomical parameters were developed for Great Basin bristlecone pine (*Pinus longaeva* D.K. Bailey) by capturing strongly contrasted microscopic images through a Confocal Laser Scanning Microscope. Environmental information embedded in wood anatomical series was analyzed in comparison with ring-width series using measures of empirical signal strength. Response functions were calculated against monthly climatic variables to evaluate climate sensitivity of cellular features (e.g., lumen area; lumen diameter) for the period 1950–2013. Calibration-verification tests were used to determine the potential to generate long climate reconstructions from these anatomical proxies. A total of eight tree-ring parameters (two ring-width and six chronologies of xylem anatomical parameters) were analyzed. Synchronous variability among samples varied among tree-ring parameters, usually decreasing from ring-width to anatomical features. Cellular parameters linked to plant hydraulic performance (e.g., tracheid lumen area and radial lumen diameter) showed empirical signal strength similar to ring-width series, while noise was predominant in chronologies of lumen tangential width and cell wall thickness. Climatic signals were different between anatomical and ring-width chronologies, revealing a positive and temporally stable correlation of tracheid size (i.e., lumen and cell diameter) with monthly (i.e., March) and seasonal precipitation. In particular, tracheid lumen diameter emerged as a reliable moisture indicator and was then used to reconstruct total March–August precipitation from 1870 to 2013. Wood anatomy holds great potential to refine and expand dendroclimatic records by allowing estimates of plant physiological adaptations to external stressors. Integrating xylem cellular features with ring-width chronologies can widen our understanding of past climatic variability (including annual extreme events) and improve the evaluation of long-term plant response to drought, especially in connection with future warming scenarios.

## Introduction

Tree-ring records are a widespread and easily accessible source of paleoclimatic information (St. George and Ault, 2014), allowing for the reconstruction of past climatic variability, from few centuries to several millennia (Hughes et al., 2011). Statistical procedures used to extract paleoclimatic signals from annual ring-widths (Jones et al., 2009) have been recently questioned, particularly after it was noted that classic tree-ring records (i.e., ring-width and maximum latewood density) used to reconstruct air temperature were showing divergence from instrumental records in the late 20th century at high latitudes (Briffa et al., 1998; D’Arrigo et al., 2008). A better mechanistic understanding of climate-growth relationships in response to climatic stressors has then become necessary on a seasonal or sub-seasonal scale (Fonti et al., 2010). Studies on seasonal dynamics of wood formation have in fact highlighted how the production of new xylem may be affected by climatic conditions occurring only in a very limited part of the year (Kirdyanov et al., 2003; Deslauriers and Morin, 2005). The quest for new dendroclimatic proxies able to capture climate-growth relationships on shorter-than-annual time scales has then pushed research efforts toward intra-annual tree-ring features and xylem anatomical structure (Seo et al., 2012; Olano et al., 2013; Nabais et al., 2014).

Among annually resolved palaeoclimatic proxies, microscopic properties of wood density (Bjorklund et al., 2015; Wood and Smith, 2015) or cell size (Pritzkow et al., 2014; Castagneri et al., 2015) represent promising approaches to improve our understanding of climate-growth relationships. Xylem anatomical traits are in fact defined by the combined effect of external climatic conditions and the internal physiological responses continuously happening inside a plant to face environmental stressors (Wimmer, 2002). Temperature is a key driver of cambial activity of conifer species, controlling the phenology of wood formation (Rossi et al., 2011) and the rate of cellular division (Gričar et al., 2014), exerting its control mostly on cell wall related features (Wang et al., 2002). At the same time, tracheids responsible for water conduction are shaped by the amount of water available at the time of their formation, hence the xylem hydraulic architecture is capable of preserving useful information for reconstructing past climatic variability (von Arx et al., 2012). In this sense, while ring-widths usually represent the outcome of an entire growing season, cellular structures (e.g., lumen area (LA), the cross sectional surface of a conducting cell) are generally affected by short-term climate conditions (Deslauriers and Morin, 2005; Martin-Benito et al., 2013) and they can be particularly effective in recording extreme events (Carrer et al., 2016).

The term “multi-proxy dendroclimatology” (McCarroll et al., 2003, 2013) describes the possibility of combining multiple tree-ring proxies, or even other geophysical and biological proxies (Neukom et al., 2010), to improve climate reconstructions. Including wood anatomy in dendroclimatic reconstructions has been hampered by the notoriously time-consuming tasks involved in processing histological samples. Eleven stem cross sections from *Larix cajanderi* Mayr. trees were used to develop cell size chronologies that could reconstruct summer temperature in north-east Siberia since 1642 (Panyushkina et al., 2003). A total of 14 cores from seven *Quercus robur* L. trees were analyzed to reconstruct minimum winter temperature from average earlywood vessel area back to 1810 in northern Poland (Pritzkow et al., 2016). A multi-proxy reconstruction of summer precipitation in Corsica that combined tree-ring, latewood and earlywood widths, cell parameters, modeled wood density, and stable carbon and oxygen isotopes of *Pinus nigra* subsp. *laricio* (Poir.) Maire (Szymczak et al., 2014) relied on 18 trees from four different sites to calculate cellular chronologies.

The application of Confocal Laser Scanner Microscopy (CLSM; Liang et al., 2013a) has improved processing and measurement of wood anatomical features, making it easier to produce century-long cellular chronologies (Liang et al., 2013b). In the western US, quantitative studies on the climatic drivers of intra-annual wood formation have been recently performed on conifer species (Hallman and Arnott, 2015; Ziaco and Biondi, 2016; Ziaco et al., 2016). It is now therefore possible to conduct cellular-based dendroclimatology on long-lived conifer species, such as the iconic bristlecone pine (*Pinus longaeva* D.K. Bailey), which has provided some of the longest annually resolved and continuous proxy records of climate (LaMarche, 1978; Hughes and Funkhouser, 2003). In this study, we used a CLSM system to measure wood anatomical parameters, and we then evaluated their effectiveness as climate proxies to improve dendrochronological reconstructions. Our objective was to develop century-long time series of wood cellular parameters from bristlecone pine to address the following questions: (i) how do cellular features compare to ring-width series in terms of the strength and temporal stability of dendroclimatic relationships?; (ii) which anatomical parameter is most sensitive to climate?; and (iii) which climatic signals can be reconstructed from wood anatomy?

## Materials and Methods

### Study Area

**FIGURE 1:**
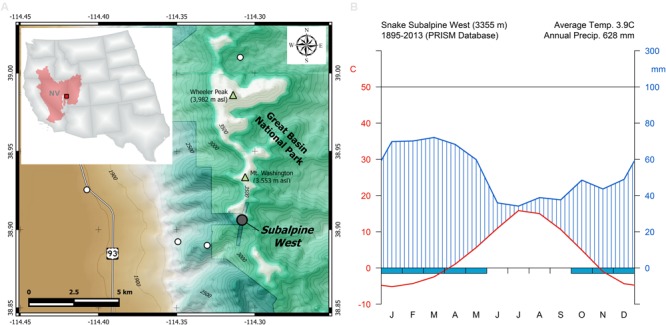
**(A)** Geographic location of the study area (red square) within the North American Great Basin (red shaded area) and the state of Nevada (NV). Sampled bristlecone pines were located in the Subalpine West site (gray circle) of the Nevada Climate-ecohydrological Assessment Network (NevCAN; other sites in this instrumented transect are shown by white circles), which is located on private land near the border of Great Basin National Park. **(B)** Walther–Lieth monthly climate diagram for the study area derived from PRISM data for the 1895–2013 period.

Wood samples were collected from bristlecone pines growing at 3355 m elevation in the Snake Range of eastern Nevada, within the Great Basin of North America (**Figure 1A**). The study site (38°54′22″N, 114°18′32″W) is part of the Nevada Climate-ecohydrological Assessment Network (NevCAN), a mountain observatory which consists of two valley-to-mountain transects established between 2010 and 2013 to collect long-term data on climate variability and its ecohydrological impacts (Mensing et al., 2013). Bristlecone pine is the dominant overstory species at the study area, with scattered individuals of limber pine (*Pinus flexilis* E.James) and Engelmann spruce (*Picea engelmannii* Parry ex Engelm.) also present. Given the short record (2011–2015) of sub-hourly climatic data recorded by the NevCAN station, monthly summaries of total precipitation, average, maximum, and minimum temperature for the site were obtained from the 4-km gridded PRISM (Parameter-elevation Relationships on Independent Slopes Model) dataset (Daly et al., 2008), and interpolated at the coordinates of the study site. During the 1895–2013 period, average annual air temperature was 3.9°C (**Figure 1B**) and mean total annual precipitation was 628 mm, showing an annual cycle dominated by cool-season precipitation, with summer being both the warmest and the driest period.

### Tree-Ring Data and Anatomical Measurements

Ten bristlecone pines (*P. longaeva* D.K. Bailey) of similar size and vigor were selected. The average diameter at breast height (1.3 m from the ground) was 38.5 ± 8.6 cm while tree height was 10.2 ± 2.0 m. Two increment cores were collected at breast height from each tree using a Swedish increment borer with inner diameter of about 4.3 mm (Grissino-Mayer, 2003). In the laboratory, these 20 cores were mounted, sanded, and crossdated using standard dendrochronological procedures (Stokes and Smiley, 1996). Ring-widths were measured to the nearest 0.001 mm with a Velmex measuring system interfaced with the program MeasureJ2X. Crossdating of ring-width measurements was checked with the *dplR* library (Bunn, 2010) for the R numerical software environment (R Core Team, 2015).

A site ring-width chronology was developed using all 20 cores extracted from the ten selected trees (**Table 1**). Measurements of anatomical parameters were performed on a subset of ten increment cores (one per each tree) using a non-destructive procedure with a Confocal Laser Scanning Microscope system that captures microscopic images directly from wood surfaces (Liang et al., 2013a). While the application of CLSM methods is well established in wood sciences (Knebel and Schnepf, 1991; Singh and Donaldson, 1999), they have been used only recently to develop time series of anatomical parameters for dendroclimatic purposes. Sample preparation followed standard procedures (Liang et al., 2013a), starting with the application of a non-Newtonian fluid (i.e., corn starch) on each mounted core to fill cell lumens and prevent cell walls from collapsing during the cutting operation (Schneider and Gärtner, 2013). Each core was then surfaced using a WSL core-microtome (Gärtner and Nievergelt, 2010), and a safranin solution was applied with a brush to the core surface to increase the contrast between cell walls and lumens. Light from a helium neon laser with wavelength of 543 nm generated by an Olympus FluoView FV300 CLSM system activated auto-fluorescence of wood. The reflected light passed through a confocal aperture where it was transformed in an electric signal, and composed into a distortion-free, well contrasted digital image at 100× magnification (Liang et al., 2013a).

**Table 1:** Correlation matrix between chronologies of ring-widths and anatomical parameters developed from bristlecone pine increment cores (20 for TR20, with two cores per tree, and 10 for all other chronologies, with one core per tree).

Tree-ring parameter	Parameter description (unit of measurement)	Year	TR20	TR10	LA	LA30	CD	LD	LW
TR20	Ring-width – 20 cores (mm)	–0.42^∗∗∗^							
TR10	Ring-width – 10 cores (mm)	–0.36^∗∗∗^	0.94^∗∗∗^						
LA	Lumen area (μm^2^)	0.41^∗∗∗^	–0.04	–0.01					
LA30	Lumen area of the 30% largest cells (μm^2^)	0.52^∗∗∗^	–0.11	–0.06	0.95^∗∗∗^				
CD	Cell radial diameter (μm; LD+DWT)	0.41^∗∗∗^	–0.12	–0.12	0.53^∗∗∗^	0.57^∗∗∗^			
LD	Lumen radial diameter (μm)	0.38^∗∗∗^	0.03	0.05	0.88^∗∗∗^	0.84^∗∗∗^	0.59^∗∗∗^		
LW	Lumen tangential width (μm)	0.67^∗∗∗^	–0.22^∗∗^	–0.18^∗^	0.85^∗∗∗^	0.83^∗∗∗^	0.54^∗∗∗^	0.71^∗∗∗^	
DWT	Double cell wall thickness (μm)	0.72^∗∗∗^	–0.33^∗∗∗^	–0.30^∗∗∗^	0.04	0.19	0.15	0.01	0.21^∗^

*The period in common among all chronologies is 1870–2013, and no detrending was applied to the individual measurement series. Correlation with time (Year) shows the presence of overall trends, which were then removed as discussed in the text. ^∗∗∗^*p* < 0.001; ^∗∗^*p* < 0.01; ^∗^*p* < 0.05.*

**FIGURE 2:**
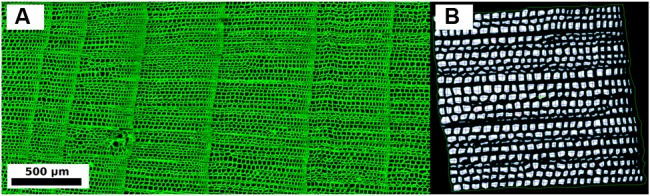
**(A)** Example of composite wood anatomy image (100-× magnification) acquired with the Confocal Laser Scanner Microscope. **(B)** Example of image with radial and tangential measurements performed on each cell within an annual tree-ring by means of the WinCELL software.

Successive images from a core were merged with Adobe Photoshop^TM^ and a shading correction was applied to remove background auto-fluorescence noise (Moëll and Donaldson, 2007). Digital CLSM produced images with black background and green cell structures (**Figure 2A**). Composite images were analyzed with the WinCELL^TM^ software (Guay, 2013). Tree-ring boundaries were visually identified on the microscopic sections and anatomical features were measured for each ring (**Figure 2B**). Since latewood was in general extremely narrow (i.e., one or two rows of cells), cell measurements were aggregated into a single annual value. Measurements were checked so that errors related to sample imperfections (e.g., resin ducts or surface breaks) could be excluded using either an automated procedure (i.e., by setting proper filters) or by the operator (Liang et al., 2013a). Annually resolved time series of anatomical parameters included average LA, the average LA of the 30% largest cells (LA30), lumen radial diameter (LD), lumen tangential width (LW), double cell wall thickness (DWT), and cell radial diameter (CD, which was given by the sum of LD and DWT) (**Table 1**). To perform dendroclimatic comparisons and considering the fact that typically many more ring-width samples can be processed in the same time required to analyze a certain number of anatomical parameters, an additional ring-width chronology was produced using only the 10 cores that were analyzed for anatomical parameters (TR10).

### Dendroclimatic Analysis

Annual tree-ring parameters, either width or anatomical features, were combined into a site chronology after removing age-related trends and other non-climatic variability (Cook and Kairiukstis, 1990). A cubic smoothing spline (Cook and Peters, 1981) was fit to each series of ring-width and cellular measurements, and indices were obtained as ratios between the measurements and the corresponding spline values. The median of all indices available for a year was used to produce the chronology value for that year, as follows:

(1) $$\begin{array}{c} {\bar{I}}_{t}=\underset{i=1\ldots \ldots ,n_{t}}{median}{\left(\frac{w_{t}}{y_{t}}\right)}_{i} \end{array}$$

where *Ī_t_* = chronology value in year *t* = median annual index; *n_t_* = number of samples in year *t*, with *n_t_* ≥ 5; *w* = crossdated ring parameter of sample *i* in year *t*; *y* = value of sample *i* in year *t* computed by fitting a cubic smoothing spline with 50% frequency response at a period of 50 years; *w_t_/y_t_* = dimensionless index value of sample *i* in year *t*. Empirical measures of dendroclimatic signals (Hughes et al., 2011), in particular average inter-series correlation (RBAR_EFF_), expressed population signal (EPS), and signal-to-noise ratio (SNR), were computed to test the strength of the environmental information embedded in the standardized chronologies using the maximum overlap of pairwise correlations (Bunn et al., 2014). The all-lag sensitivity of the chronologies was quantified by the Gini coefficient (Biondi and Qeadan, 2008).

Response functions between tree-ring chronologies and monthly values of average, maximum, and minimum air temperature and total precipitation from the PRISM dataset were computed using the R package *treeclim* (Zang and Biondi, 2015). The period 1950–2013 was selected to avoid climate data uncertainty, mostly caused by very sparse coverage during earlier years (1895–1949) in the western USA (Guttman and Quayle, 1996). Dendroclimatic relationships over the water-year window, from the previous October to the current September, were tested using 1000 bootstrapped samples. Moving response functions (Biondi, 1997) for a 30-year sliding interval allowed for evaluating the stability of dendroclimatic relationships.

Seasonal correlations between climate and tree-ring chronologies were computed by means of partial correlation for season lengths from three to six months, using the approach of (Meko et al., 2011) as implemented in *treeclim* (Zang and Biondi, 2015). Skill of ring-width and anatomical proxies for reconstructing past climate was tested by performing ordinary least squares regression for the most significant seasonal correlation that emerged from partial correlation analysis between ring-width and anatomical parameters against precipitation. The period 1950–2013 was used for a split calibration-verification exercise using the reduction of error (RE), the coefficient of efficiency (CE), and the Durbin–Watson test (Biondi et al., 2008).

## Results

### Tree-Ring and Wood Anatomy Chronologies

**FIGURE 3:**
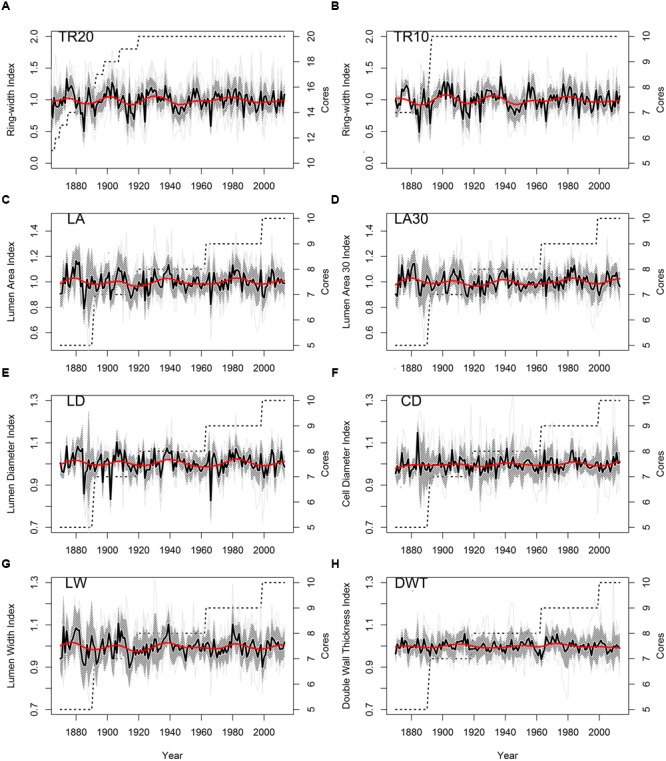
**Standardized chronologies (1870–2013) of annual ring-width and anatomical parameters of bristlecone pine (light gray lines, individual series; shaded area, ± 1 standard deviation; red line, 30-year cubic spline; dashed line, number of cores). (A)** Ring-width (20 cores); **(B)** ring-width (10 cores); **(C)** lumen area (LA); **(D)** LA of the 30% largest cells; **(E)** lumen radial diameter; **(F)** cell radial diameter; **(G)** lumen tangential width; **(H)** double wall thickness. Note the different scales of the *y*-axes.

Stem ages of sampled bristlecone pines ranged from 121 to 240 years. Crossdating was simplified by the absence of false or missing rings, and by the presence of several “pointer years” (e.g., 1966, 1924, 1902, 1884). In all cores the 1902 earlywood showed the typical features of “frost rings” (Schweingruber, 2007), indicative of a late freezing event in the spring of that year. In the 1884 latewood an early-frost event was visible in about half of the cores. Without detrending of individual series, median chronologies of ring-widths (TR20 and TR10) showed an overall declining pattern, whereas median chronologies of anatomical parameters (LA, LA30, LD, CD, LW, DWT) presented an opposite trend, in particular DWT and LW (**Table 1**). Most anatomical parameters were not correlated with ring-widths (either TR20 and TR10), with the only exception of LW and DWT (**Table 1**). Standardized chronologies started in 1870, the first year with at least five overlapping anatomical samples, and ended in 2013, for a total length of 144 years (**Figures 3A–H**). Anatomical chronologies presented similar or lower first-order autocorrelation (ar1) compared to ring-width series. The parameters LW and DWT again behaved differently, presenting the highest ar1 values, while CD had the lowest one (**Table 2**).

**Table 2:** Summary of ring-width and anatomical chronologies for the 1870-2013 period (144 years).

Tree-ring parameter	ar1	StDev	G	RBAR_EFF_	EPS	SNR
TR20	0.284	0.141	0.079	0.336	0.905	9.476
TR10	0.308	0.147	0.082	0.320	0.818	4.500
LA	0.289	0.069	0.038	0.207	0.672	2.046
LA30	0.316	0.060	0.034	0.200	0.661	1.953
LW	0.392	0.042	0.023	0.108	0.486	0.947
LD	0.119	0.044	0.024	0.298	0.768	3.317
DWT	0.346	0.022	0.012	0.035	0.221	0.284
CD	–0.014	0.031	0.017	0.186	0.641	1.787

*For acronyms see **Table 1**. ar1, first-order autocorrelation of standardized chronologies; StDev, standard deviation of standardized chronologies; G, Gini coefficient of standardized chronologies; RBAR_EFF_, between-series correlation; EPS, expressed population signal; SNR, Signal-to-Noise ratio.*

Common environmental signals recorded by the tree-ring chronologies decreased from ring-width to wood anatomy parameters. Measures of empirical signal strength (**Table 2**) for the site ring-width chronology (TR20) were in the same order of magnitude of other *P. longaeva* chronologies (LaMarche and Stockton, 1974). Signal strength in TR10 was also high and values of RBAR_EFF_, SNR, and EPS exceeded the minimum thresholds suggested for sufficiently replicated chronologies (Wigley et al., 1984). Among cellular features, LD showed the highest RBAR_EFF_, SNR, and EPS (**Table 2**). Parameters linked to plant’s hydraulic performance (LA and LA30) and CD (a combined parameter including LD and DWT) had lower SNR and EPS, but higher Gini coefficients than other anatomical chronologies (**Table 2**). Noise was predominant (SNR < 1) in chronologies of lumen width (LW) and DWT, and both RBAR_EFF_ and EPS dropped to extremely low values (**Table 2**). These two anatomical parameters were therefore excluded from further dendroclimatic calibration.

### Dendroclimatic Relationships

**FIGURE 4:**
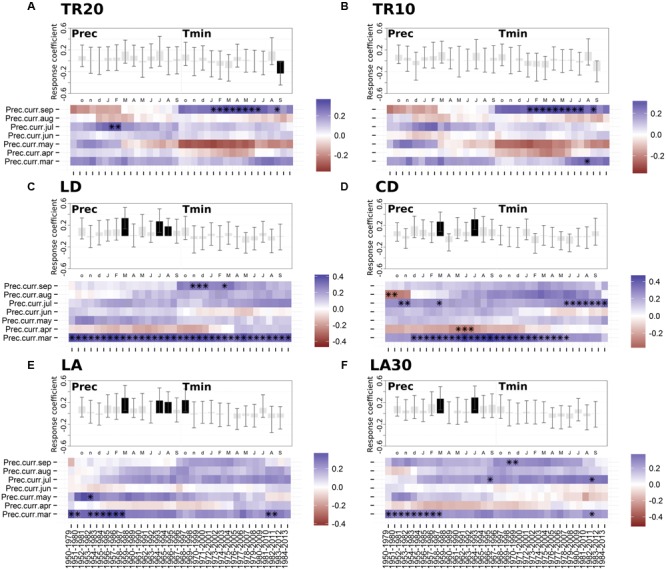
**Static (upper graph; significant months are shown in black) and moving (lower graph; significant months are marked by black asterisks) response functions of annual tree-ring chronologies against minimum air temperature and monthly precipitation for the period 1950-2013. (A)** Ring-width (20 cores); **(B)** ring-width (10 cores); **(C)** lumen radial diameter; **(D)** cell radial diameter; **(E)** lumen area (LA); **(F)** LA of the 30% largest cells. Both temperature and precipitation, from previous October to current September (lowercase letters, previous year months; uppercase letters, current year months), were used in the upper graph (vertical bars indicate 95% confidence interval), while precipitation from March to September and a 30-year moving interval was used in the lower graph. For acronyms see **Table 1**.

Dendroclimatic response functions highlighted the presence of a general positive correlation with current year precipitation in 1950–2013 (**Figure 4**). All the anatomical parameters showed a significant correlation with March and July precipitation (**Figures 4C–F**). Radial size of cell lumens (LD) and average LA were also positively related to August precipitation (**Figures 4C,E**). Only the ring-width chronology based on 20 cores (TR20) showed a significant inverse relationship with September minimum temperature (**Figure 4A**), while LA presented a positive correlation with previous October minimum temperature (**Figure 4E**).

Since no significant relationships emerged between anatomical parameters and mean, maximum, or minimum temperature (with the only exception of the above mentioned relationships between LA and previous October temperature), moving response functions were computed only for precipitation. The importance of March and July precipitation for shaping wood cellular features was particularly evident for lumen and cell diameter (CD; **Figures 4C–D**). March was stronger and more temporally stable in LD and CD chronologies compared to other anatomical parameters (e.g., LA). Response to July moisture remained positive from 1950 to 2013, even though it seemed to increase in LD, CD, and LA in the last 35–40 years (**Figures 4C–E**). Approximately in the same period, the precipitation signal in April and June reversed for both LD and CD, showing a positive effect in April and a negative effect in June.

**FIGURE 5:**
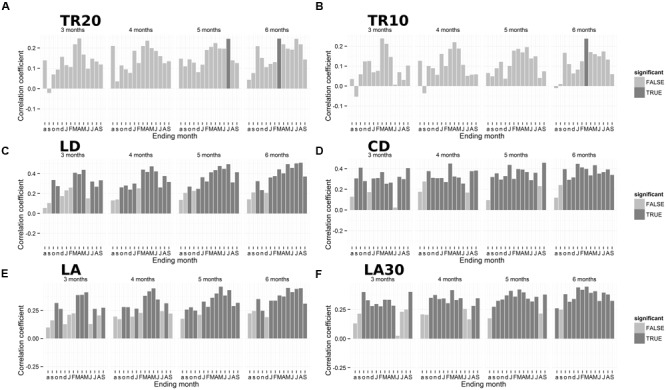
**Plot of seasonal correlations between annual tree-ring parameters and total precipitation for 3, 4, 5, and 6-month seasons. (A)** Ring-width (20 cores); **(B)** ring-width (10 cores); **(C)** lumen radial diameter; **(D)** cell radial diameter; **(E)** lumen area (LA); **(F)** LA of the 30% largest cells. Dark gray bars represent significant coefficients using a 95% confidence interval. Lowercase letters, previous year months; uppercase letters, current year months. Note the different scales of the *y*-axes. For acronyms see **Table 1**.

Partial correlation analysis for the period 1950–2013 indicated a positive seasonal precipitation signal for anatomical chronologies (**Figure 5**). Ring-width chronologies presented a significant seasonal relationship with total precipitation over the previous autumn and winter (October–March), which emerged in both TR20 and TR10 (**Figures 5A–B**). Anatomical parameters LD and CD showed a higher correlation with spring-summer precipitation during the current year. Lumen diameter (LD) was highly correlated with March–August precipitation (*r* = 0.51; **Figure 5C**), while CD was positively linked to total precipitation from May to September (*r* = 0.46; **Figure 5D**). LA (**Figure 5E**) and the average size of the top 30% largest cells (**Figure 5F**) were correlated to winter and spring precipitation, LA from January to May (*r* = 0.46) and LA30 from previous October to current March (*r* = 0.45).

**FIGURE 6:**
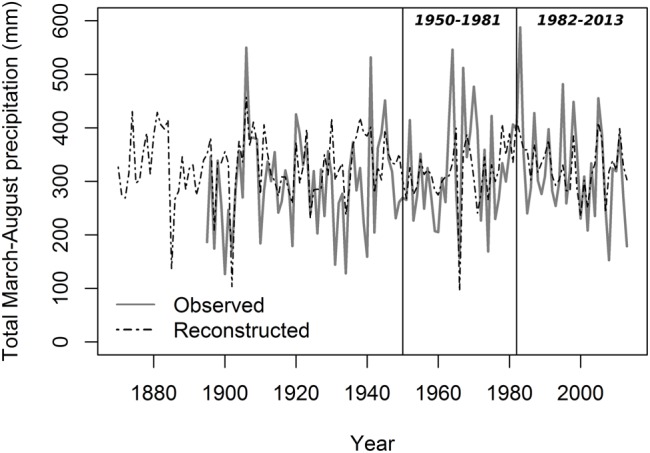
**Reconstructed total March–August precipitation from 1870 to 2013 using lumen diameter.** Gray solid line, PRISM data; black dashed line, reconstructed series. Vertical lines define the two periods (1950–1981 and 1982–2013) used for the split calibration/verification exercise.

Lumen diameter emerged as the best potential proxy for reconstructing past seasonal precipitation from 1870 to 2013 (**Figure 6**; **Table 3**). The regression model between LD and total March–August precipitation explained about one-third of the total variance (*R*^2^ = 0.31), and verification indices (both RE and CE > 0) were satisfactory for the two split calibration/verification periods (1950–1981 and 1982–2013; **Table 3**). Verification indices between CD and May–September total precipitation were also acceptable (in particular for the period 1982–2013) but the model built using CD explained less variance (*R*^2^ = 0.23) and the Durbin–Watson test pointed to higher residual autocorrelation (**Table 3**). Other anatomical parameters (LA and LA30) had good verification statistics, respectively, for the period 1950–1981 and 1982–2013, but the overall model explained a limited amount of variance (*R*^2^ = 0.20 and 0.19 for LA and LA30). Ring-width chronologies could not be used to reconstruct past variability of total October–March precipitation, as the verification statistics and model’s R^2^ were extremely low for both TR20 and TR10 (**Table 3**).

**Table 3:** Calibration-verification statistics for dendroclimatic reconstructions of precipitation using ring-width and selected anatomical parameters in 1950-2013.

		1950–1981			1982–2013			Whole model		
Tree-ring parameter	Climate season	RE	CE	DW	RE	CE	DW	Intercept	Slope	*R*^2^
TR20	Oct–Mar	–0.004	–0.093	2.010	0.053	–0.045	2.527	112.45	250.10	0.06
TR10	Oct–Mar	–0.085	–0.182	2.014	0.064	–0.034	2.572	126.67	235.16	0.06
LD	Mar–Aug	0.109	0.108	1.879	0.362	0.362	1.933	-976.47	1297.30	0.31
CD	May–Sep	0.082	0.072	1.555	0.369	0.360	1.882	–1255.57	1479.37	0.23
LA	Jan–May	0.254	0.252	1.905	–0.008	–0.012	3.05	-504.48	850.36	0.20
LA30	Oct–Mar	0.199	0.127	2.395	0.269	0.193	2.652	-616.93	979.01	0.19

*Verification statistics RE (Reduction of Error), CE (Coefficient of Efficiency) and DW (Durbin–Watson test) are shown for the two split periods, 1950-1981 and 1982-2013 (calibration/verification and vice versa).*

## Discussion

### Ring-Width and Cellular Chronologies

These 144-year long, crossdated, cellular chronologies represent a first attempt at developing proxy climate records from anatomical features of Great Basin bristlecone pine. Similarly long time series of anatomical parameters (e.g., cell wall thickness; vessel area) have already been generated in Canada and Europe for hardwood (Tardif and Conciatori, 2006; Fonti and García-González, 2008) and conifer species, including *Pinus nigra* Arn. (Martin-Benito et al., 2013), *Pinus sylvestris* L. (Eilmann et al., 2009; Seo et al., 2012; Pritzkow et al., 2014), and *Picea abies* Karst. (Castagneri et al., 2015), *Picea mariana* Mill. (Wang et al., 2002), and *Picea glehnii* Mast. (Yasue et al., 2000). To our knowledge no such chronologies exist in the western US, despite the presence of several long-lived conifer species. In particular, the importance of *P. longaeva* for paleoclimatic reconstructions can hardly be overstated, given the species’ property of attaining stem ages in excess of 5000 years (Currey, 1965).

Xylem cellular features were not correlated with ring-width, with the only exception of the cell wall thickness and tangential LW. Our results then suggest that wood anatomy of bristlecone pine carries a specific environmental signal, which is not a replicate of the signal encoded in ring-width series, but rather represents a source of additional paleoclimatic information (Eckstein, 2004). In our dataset, ar1 of most anatomical parameters was lower than for ring-width chronologies, as intra-annual tracheids are largely affected by environmental conditions at the time of their formation, in particular the tree hydration status (Steppe et al., 2015). Reduced ar1 in anatomical time series (i.e., tracheid and vessel size) compared to ring-width series has been in fact reported in conifers (Pritzkow et al., 2014; Pacheco et al., 2016) and in hardwoods (García-González and Eckstein, 2003; Fonti and García-González, 2008). The values of ar1 for the chronologies of DWT and LW can be explained considering the seasonal dynamics of wood formation. Cell wall thickness can present high temporal autocorrelation (Martin-Benito et al., 2013), likely because of how previously stored carbon pools contribute to the current year growth (Hansen and Beck, 1990), while the tangential diameter of developing xylem cells remains almost constant during the elongation phase (Cuny et al., 2014).

The EPS of LD and CD was comparable with values found in ring-widths of bristlecone pine growing at the treeline in the southwest US (Salzer et al., 2014), yet lower than the common signal in ring-widths (TR10). Weaker empirical signal strength, in particular inter-series correlation, for cellular chronologies compared to ring-width series, can be the consequence of lower inter-annual variability in microscopic features of wood (Olano et al., 2012; Liang et al., 2013b; Pritzkow et al., 2014). On the other hand, a relatively weak common signal in annual anatomical parameters can correspond to significant relationships with intra-annual environmental variables (Yasue et al., 2000). Information recorded in xylem anatomy may in fact reflect internal physiological processes (Fonti and García-González, 2008) rather than limiting factors exerted over the entire growing season (Eckstein, 2004).

### Potential for Dendroclimatic Reconstructions

Climate-growth relationships at the cellular level have been investigated using undetrended series (Liang et al., 2013b) as well as standardized chronologies (Bryukhanova and Fonti, 2013). Standardization is a critical issue in dendrochronology (Hughes et al., 2011), and its importance has become evident even for xylem anatomy, especially when the length of individual series exceeds multiple decades (Pritzkow et al., 2014, 2016; Carrer et al., 2015). Our cellular chronologies of bristlecone pine were characterized by an age-related trend, which made standardization useful. Cellular parameters were better dendroclimatic proxies than ring-width chronologies when detrending techniques commonly applied to ring-width series were used. In our study, dendroclimatic response functions of annual tree-ring parameters showed a dominant precipitation signal, especially for anatomical parameters LD, CD, and LA, and a very weak temperature signal. This is in contrast with the positive response to temperature and the weak correlation with precipitation found by (Salzer et al., 2009) at the Snake Range. Such discrepancy could be related to the age of the individuals sampled in these studies, and it reflects the complexity of climatic responses near treeline (Jolly et al., 2005), which can vary even at very short spatial scales, especially for *P. longaeva* (Salzer et al., 2014).

The ecological meaning of climate-anatomy relationships can be better understood if integrated with information on xylem phenology (e.g., the timing of wood formation) and intra-annual dynamics of stem growth obtained by repeated microcoring and dendrometer records (Rossi et al., 2012; Cocozza et al., 2016; Pacheco et al., 2016). In a study recently conducted at the same site (Ziaco et al., 2016), production of new tracheids in bristlecone pine during two consecutive years (2013–2014) started at the beginning of June, immediately after snowmelt, peaked in late June, and wood formation ended in mid-September. Cellular measurements showed that mean air temperatures between 6°C and 7°C were linked with the production of new tracheids, while dendrometer records highlighted the influence of soil temperature for snowpack thawing and subsequent plant-soil water exchanges (Ziaco et al., 2016).

Conifer species growing in drought-prone environments escape hydraulic failure (e.g., cavitation) because their reduced conduit size (e.g., LA, LD) and enhanced cell wall thickness (DWT) can resist higher negative water pressures (Hacke et al., 2001). The positive and temporally stable precipitation signal found in cellular chronologies of LD, CD, and LA can be divided in a winter (i.e., March) and summer component (i.e., July–August), which most likely relate to water-use strategies throughout the growing season. March precipitation at the study area is important to recharge soil water, providing trees with moisture needed in the first phases of xylogenesis for maintaining cellular turgor (Turcotte et al., 2009). Summer precipitation relieves water stress and increases the margins for hydraulic safety, leading to the formation of larger cellular elements (Martin-Benito et al., 2013; Ziaco et al., 2014; Pacheco et al., 2016). It should also be noted that at high elevation (i.e., near treeline), reduced lumen sizes can also be interpreted as an adaptation of bristlecone pine to reduce the risk of freezing-induced embolisms (Pittermann and Sperry, 2003). These ecophysiological processes make it possible to use the inter-annual variability of tracheid size as a reliable descriptor for tree hydraulic adjustments (Bryukhanova and Fonti, 2013).

The seasonal climate-growth relationships uncovered by partial correlation analysis suggest that in these arid and semi-arid environments water availability plays a crucial role on the xylem anatomy of bristlecone pine. An adequate supply of moisture is needed during all phases of cellular differentiation, especially in dry environments (Vieira et al., 2014), and the anatomical structures formed throughout the growing season are often related to intra-annual climatic episodes (Deslauriers and Morin, 2005), including the most extreme ones (Carrer et al., 2016). Water availability also controls carbon consumption and modulates internal distribution of non-structural carbohydrates (Sevanto et al., 2014), ultimately driving mortality patterns of entire forest ecosystems (McDowell et al., 2008). Different time scales of physiological processes driven by water availability might therefore explain the prolonged seasonal response of xylem anatomy identified in this study on *P. longaeva*. Even the negative correlation between ring-width chronologies and September temperature could reflect an indirect response to moisture, as suggested by the positive correlation between TR20 and September precipitation that emerged after 1970 (**Figure 4A**). At high elevations, daily temperature variability is regulated by incoming solar radiation, therefore cloudy skies are associated with a higher chance of precipitation, limited evapotranspiration, and lower air temperatures (Biondi and Rossi, 2015). Water is a key driver of cambial resumption in the dry season (Camarero et al., 2010), and trees could therefore use the additional moisture available in late August-early September to delay the cessation of wood production.

Bristlecone pine, thanks to its remarkable longevity, has provided reliable reconstructions of temperature and precipitation in the western US over the last millennium, through chronologies based on ring-widths (Routson et al., 2011; Tolwinski-Ward et al., 2015) or stable isotopic ratios (Leavitt, 1994; Bale et al., 2011). In this study, ring-width and anatomical chronologies performed differently as dendroclimatic proxies for reconstructing past seasonal precipitation. Chronologies of LD, CD, and LA had empirical signal strength statistics lower than both TR20 and TR10, but provided better precipitation proxies. Our reconstruction of total March–August precipitation based on LD of tracheids passed typical calibration/verification tests, even if developed using a limited amount of samples (10 cores). This result suggests that dendroclimatic information recorded in cellular anatomy of bristlecone pine could be used to refine our understanding of past climatic variability in the Great Basin, including the frequency of intra-annual extreme events or the occurrence and duration of severe drought episodes.

## Conclusion

The potential of xylem anatomical features as dendroclimatic proxies in a semi-arid environment of the Western US was investigated in comparison with traditional ring-width chronologies. Relationships between wood anatomy and climate were temporally stable and different from ring-widths, suggesting that tracheid size, in particular LD and CD, can provide a valid representation of plant physiological adaptations to external stressors. This study is a first step toward a “multi-proxy” approach (McCarroll et al., 2003; Gagen et al., 2006) to dendroclimatic studies in the Great Basin, so that wood cellular features can be integrated in long reconstructions of past climatic variability. Adding wood anatomical proxies to the suite of records that is recoverable from millennia-old trees holds substantial scientific value. Our findings suggest that cellular features of Great Basin bristlecone pine incorporate a seasonal signal linked to precipitation. Since this kind of climatic information is unusual for high elevation bristlecone pine, the possibility exists to expand multi-millennia climatic records based on this and other conifer species of the western US, whose lifespan often exceeds several centuries.

## Author Contributions

EZ and FB conceived the research design and the sampling procedures. EZ conducted the sample collection. EZ performed the measurements and the elaboration of raw data in collaboration with IH. FB and EZ performed the dendroclimatic analysis, and developed hypotheses with IH. EZ and FB wrote the manuscript, and IH contributed to the final version.

## Conflict of Interest Statement

The authors declare that the research was conducted in the absence of any commercial or financial relationships that could be construed as a potential conflict of interest.
